# Integrating multidimensional nociceptive-related cortical features for unsupervised assessment of anesthesia states in rats

**DOI:** 10.1016/j.isci.2026.116152

**Published:** 2026-05-28

**Authors:** Fengrui Zhang, Wenqian Zhou, Wen Liang, Ruoyu Wang, Libo Zhang, Xiao Zhang, Meizi Liu, Tong Li, Lupeng Yue, Li Hu

**Affiliations:** 1 State Key Laboratory of Cognitive Science and Mental Health, Institute of Psychology, Chinese Academy of Sciences, Beijing 100101, China; 2 Department of Psychology, University of Chinese Academy of Sciences, Beijing 100101, China; 3 Department of Psychology, Shandong Second Medical University, Weifang 261000, China; 4 Department of Electrical and Computer Engineering, University of California, San Diego, San Diego, CA 92093, USA; 5 School of Computer Science and Engineering, The University of New South Wales, Sydney, NSW 2052, Australia; 6 Institute for Diabetes, Obesity and Metabolism, University of Pennsylvania, Philadelphia, PA 19104, USA; 7 Department of Medicine, Washington University School of Medicine, St. Louis, MO 63110, USA; 8 Expedia Group, Seattle, WA 98119, US

**Keywords:** Biological sciences, Neuroscience, Artificial intelligence, Machine learning

## Abstract

Accurate anesthesia monitoring remains challenging because current approaches primarily assess consciousness while overlooking nociceptive processing. Although electroencephalography (EEG)-based metrics such as permutation entropy (PE) and permutation cross-mutual information (PCMI) are widely used, nociceptive-evoked cortical responses, especially gamma-band oscillations (GBOs), a robust index of nociceptive intensity, are rarely incorporated into anesthesia assessment. Here, we recorded electrocorticography from 23 rats under isoflurane anesthesia with nociceptive laser stimulation during both induction and emergence. We extracted GBOs, PE, and PCMI to evaluate their sensitivity to anesthesia states. GBOs and PE tracked nociceptive-related changes during induction, whereas PCMI was more sensitive during emergence. Integrating these multidimensional features, an unsupervised *k*-means framework identified four latent states: awake, shallow anesthesia, moderate anesthesia, and burst suppression. These findings establish nociceptive-evoked cortical responses as label-free markers and provide a scalable foundation for real-time, closed-loop anesthesia monitoring that jointly assesses consciousness and analgesia.

## Highlights

• Nociceptive-evoked cortical signals track anesthesia-state transitions
• GBOs, PE, and PCMI capture distinct neural dimensions of anesthesia
• Unsupervised clustering identifies four latent anesthesia states
• Integrated features enable label-free, brain-state stratification

## Graphical abstract

**Figure:**
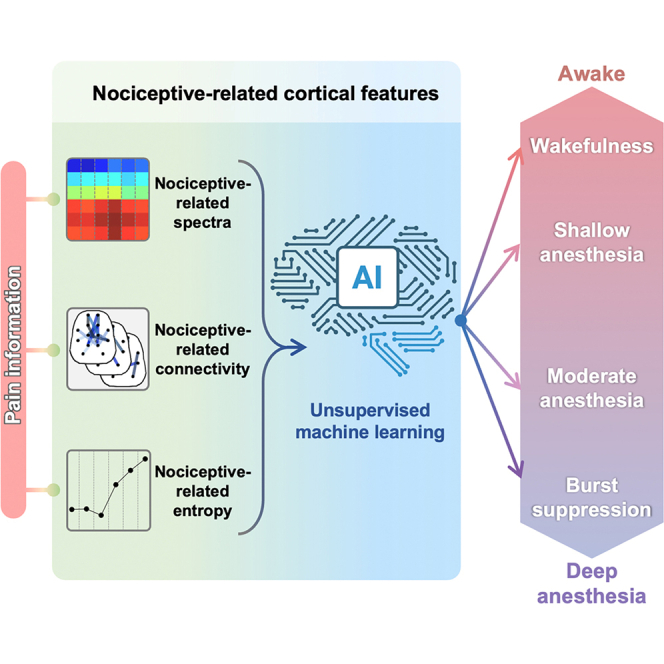

## Introduction

General anesthesia is indispensable in modern medicine, ensuring patient comfort and safety by alleviating surgery-induced discomfort, pain, and related complications. Effective anesthesia monitoring is essential for maintaining an appropriate anesthesia depth and preventing intraoperative awareness or excessive suppression of vital functions. Currently, anesthesia monitoring primarily relies on electroencephalography (EEG)-based metrics,^1^^,^^2^^,^^3^ including spontaneous EEG spectral power,^4^ auditory evoked potentials,^5^ bispectral index (BIS),^6^ and EEG complexity and connectivity measures,^7^ such as permutation entropy (PE)^8^ and permutation cross-mutual information (PCMI).^9^ Although these EEG-based metrics effectively capture the loss and recovery of consciousness, they lack direct sensitivity to nociceptive processing, which limits their sensitivity in adequately capturing the analgesic component of anesthesia. This limitation contributes to the long-standing clinical challenge of achieving an optimal balance between sedation and analgesia. Integrating nociceptive-related neural responses into anesthesia monitoring frameworks may offer a path toward resolving this gap.

Among various nociceptive-related neural responses, cortical oscillations in the gamma frequency range (gamma-band oscillations, GBOs) have emerged as a robust electrophysiological signature of pain processing across multiple species.^10^^,^^11^^,^^12^ In humans, GBOs encode perceived pain intensity independently of the physical stimulus intensity and reliably track perceived pain, even when stimulus salience is markedly reduced by repetition.^13^ Furthermore, GBOs correlate with perceived pain intensity at both within- and between-subjects levels but not with similarly salient non-nociceptive stimuli, such as tactile, auditory, and visual inputs.^10^^,^^14^^,^^15^ In rodents, recent causal evidence further demonstrates that nociceptive-evoked GBOs preferentially encoding pain intensity are generated by parvalbumin-positive interneurons in the primary somatosensory cortex.^16^^,^^17^ Collectively, these findings identify GBOs as objective and reliable neural correlates of pain perception, making them promising candidates for the development of pain-sensitive anesthesia monitoring systems capable of dynamically assessing analgesic states during surgery.

However, translating nociceptive-related neural responses into practical monitoring tools remains challenging. Under deep anesthesia, cortical responsiveness is markedly suppressed, resulting in low-amplitude and variable signals that are highly dependent on individuals, time, anesthetic depth, and so on.^18^^,^^19^ These conditions require analytical frameworks that can extract reliable patterns from high-dimensional, weak, and non-stationary signals. Unsupervised machine learning is well suited to address this challenge, as it can integrate high-dimensional signals, identify latent features within complex datasets, and adaptively classify brain states based on nociceptive-related cortical features.^20^^,^^21^ By leveraging these strengths, it becomes possible to construct monitoring systems that incorporate nociceptive-related cortical features with well-established sedation indices, thereby providing a more comprehensive assessment of anesthesia depth.

In this study, we propose an anesthesia monitoring approach to automatically identify anesthesia states based on multidimensional nociceptive-evoked cortical features. Electrocorticography (ECoG) data were collected from 23 rats (12 males and 11 females) undergoing general anesthesia induced by isoflurane, during both induction and emergence phases, while receiving nociceptive laser stimuli. We systematically assessed the sensitivity of nociceptive-evoked cortical responses as neural indicators to anesthesia states. An unsupervised machine learning model was developed to identify anesthesia states by integrating nociception-evoked cortical responses and established sedation-related EEG indicators (i.e., PE and PCMI). Our results demonstrate that incorporating nociceptive information substantially enhances anesthesia state classification performance, establishing a computational foundation for developing advanced monitoring tools capable of simultaneously tracking both consciousness and nociceptive processing.

## Results

### A laser-pain model during anesthesia

**Figure 1:**
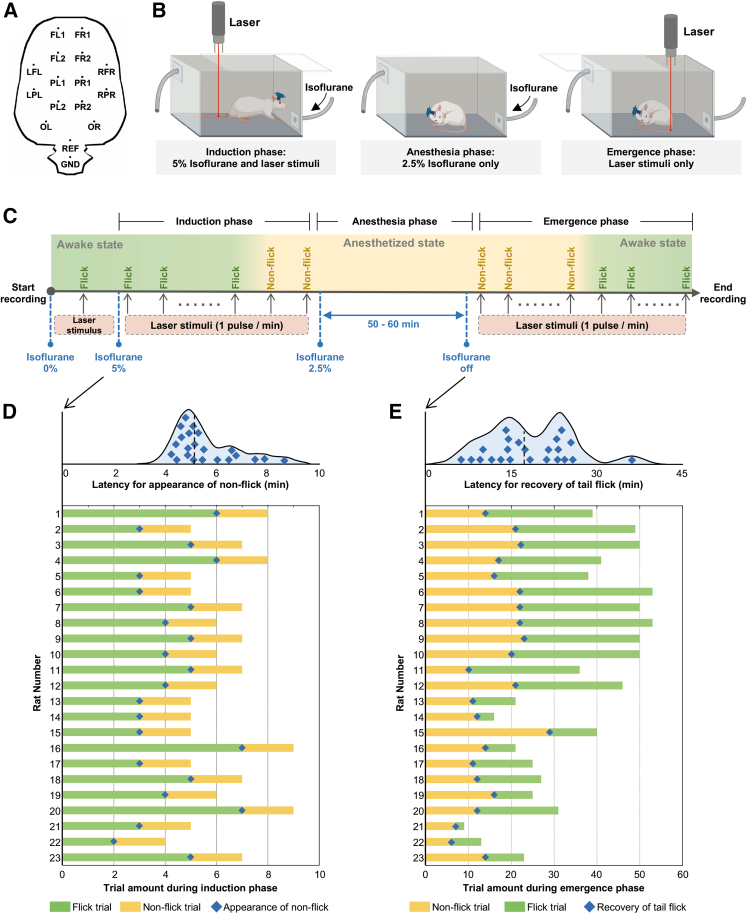
Experimental design and behavioral results The figure legend can be all one paragraph and describe the images (A), graphs (B), and plots (C), etc., together. (A) Electrode placement for ECoG recording. (B) Schematic of the experimental setup and procedures during the induction, anesthesia, and emergence phases. (C) Diagram of rat ECoG recording during isoflurane-induced general anesthesia with nociceptive laser stimuli. (D) Distribution of induction time (top) and behavior responses to each laser stimulus (bottom) of all 23 rats during the induction phase. Time 0 indicates the onset of anesthesia induction. (E) Distribution of tail-flick recovery time (top) and behavior responses to each laser stimulus (bottom) of all 23 rats during the emergence phase. Time 0 indicates the offset of isoflurane administration.

**Figure 2:**
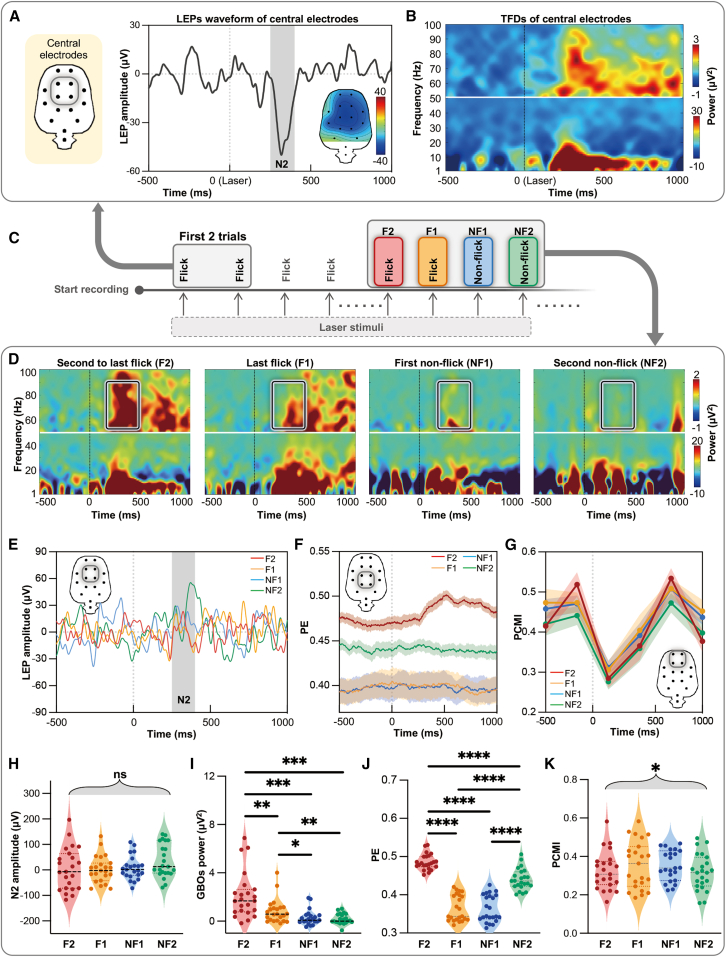
Nociceptive-evoked neural responses discriminate anesthesia states during the induction phase (A) Group-level LEP waveforms recorded from central electrodes (i.e., PL2, PR2, PL1, and PR1) and scalp topographies of LEP-N2 wave for the first two laser stimuli. (B) Group-level TFDs of nociceptive-evoked neural responses from the same electrodes for the first two laser stimuli. (C) Diagram of selected trials for subsequent analyses during the induction phase. (D and E) Group-level TFDs (D) and LEPs (E) of nociceptive-evoked neural responses for the four selected trials (i.e., F2, F1, NF1, and NF2) at central electrodes. (F) Group-level PE computed from parietal electrodes (i.e., PL1, PR1, PL2, and PR2) for the four selected trials (shaded area indicates the SEM). (G) Group-level PCMI computed from frontal electrodes (i.e., FL1, FR1, FL2, and FR2) for the four selected trials (shaded area indicates the SEM). (H–K) Statistical comparisons of (H) LEP-N2 amplitude (250–400 ms post-stimulus), (I) GBO power (55–99 Hz, 100–500 ms post-stimulus), (J) PE (0–1,000 ms post-stimulus), and (K) PCMI (0–500 ms post-stimulus) across the four selected trials (*n* = 23 rats). Statistical analyses were conducted using one-way repeated-measures ANOVA followed by post-hoc paired-sample *t* tests with Bonferroni correction. ∗*p* < 0.05; ∗∗*p* < 0.01; ∗∗∗*p* < 0.001; ∗∗∗∗*p* < 0.0001; ns, no significance. F2, the second-to-last flick trial; F1, the last flick trial; NF1, the first non-flick trial; NF2, the second non-flick trial. See also Table S1–S4.

To capture nociceptive responses across the transition from wakefulness to deep anesthesia, we developed a rat electrophysiological model with nociceptive laser stimuli under graded levels of isoflurane. Nociceptive laser stimuli were delivered to the tail during both the induction and emergence phases of anesthesia (Figures 1A and 1B). For each stimulus, tail-flick behaviors, a well-established nociceptive reflex used to assess analgesic efficacy,^22^ were recorded along with ECoG signals (Figure 1C). During the induction phase, tail-flick responses were initially observed in response to laser stimuli but ceased after approximately 3–9 min of isoflurane exposure (Figure 1D). During the emergence phase, tail-flick responses were initially absent for 5–30 min and then gradually reappeared as the animals recovered from anesthesia (Figure 1E). We next examined the electrophysiological features of this laser-pain model by extracting laser-evoked responses in both the time (Figure 2A) and time-frequency domains (Figure 2B). Consistent with previous studies,^10^^,^^16^^,^^17^^,^^23^ evident LEP-N2 wave and GBOs were observed at central electrodes (e.g., FL2, FR2, PL1, and PR1) after laser stimuli. These results validate the effectiveness of the laser-pain model, upon which all subsequent analyses were based.

### Laser-evoked cortical responses track anesthesia state transitions during induction

To examine whether nociceptive-related neural features could serve as indicators of anesthesia state transitions during induction, we evaluated the ability of all four estimated electrophysiological measures, i.e., LEP-N2 amplitude, GBOs, PE, and PCMI, to discriminate different anesthesia states. Specifically, we analyzed four consecutive trials surrounding the loss of tail-flick responses (Figure 2C), i.e., the second-to-last flick (F2), the last flick (F1), the first non-flick (NF1), and the second non-flick (NF2), as these trials represent the transition from wakefulness to anesthesia. One-way repeated-measures ANOVA revealed significant effects of trial type on GBOs (F_(3, 66)_ = 17.39, *p* < 0.001, Figure 2D), PE (F_(3, 66)_ = 209.7, *p* < 0.0001, Figure 2F), and PCMI (F_(3, 66)_ = 3.447, *p* = 0.0215, Figures 2G and 2K) but not on LEP-N2 amplitude (F_(3,66)_ = 1.442, *p* = 0.244, Figures 2E and 2H; Tables S1–S7). Post-hoc pairwise comparisons further revealed that GBOs and PE could distinguish subtle changes across the four trial types (Figures 2I and 2J), while PCMI failed to do so. Collectively, these results indicated that while LEP-N2 amplitude is insensitive to fluctuations in anesthesia state shifting, GBOs and PE effectively track subtle state changes during anesthesia induction and motor responsiveness. In contrast, PCMI provides a less precise measure of anesthesia state changes.

### Laser-evoked cortical responses discriminate anesthesia states during emergence phase

**Figure 3:**
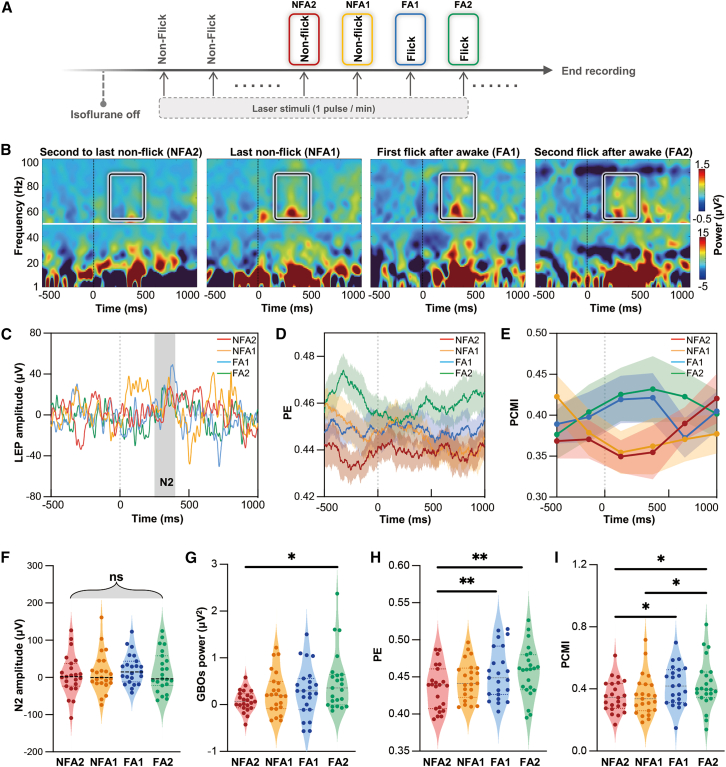
Nociceptive-evoked neural responses are also able to discriminate anesthesia states during the emergence phase (A) Diagram of the four selected trials for subsequent analyses during the emergence phase: NFA2, NFA1, FA1, and FA2. (B and C) Group-level TFDs (B) and LEPs (C) of nociceptive-evoked neural responses for the four selected trials at central electrodes. (D) Group-level PE at parietal electrodes for the four selected trials (shaded area indicates the SEM). (E) Group-level PCMI at frontal electrodes for the four selected trials (shaded area indicates the SEM). (F–I) Statistical comparisons of (F) LEP-N2 amplitude (250–400 ms post-stimulus), (G) GBO power (55–99 Hz, 100–500 ms post-stimulus), (H) PE (0–1,000 ms post-stimulus), and (I) PCMI (0–500 ms post-stimulus) across the four selected trials (*n* = 23 rats). Statistical analyses were conducted using one-way repeated-measures ANOVA followed by post-hoc paired-sample *t* tests with Bonferroni correction. ∗*p* < 0.05; ∗∗*p* < 0.01; ns, no significance. NFA2, the second-to-last non-flick trial; NFA1, the last non-flick trial; FA1, the first flick trial after awakening; FA2, the second flick trial after awakening. See also Table S5–S8.

To further examine whether nociceptive-related neural features could reliably track changes in anesthesia state and nociceptive processing during the emergence phase, we analyzed four consecutive trials surrounding the recovery of tail-flick responses (Figure 3A), i.e., the second non-flick before awakening (NFA2), the last non-flick before awakening (NFA1), the first flick after awakening (FA1), and the second flick after awakening (FA2), representing the transition from anesthesia to wakefulness. Similarly, LEP-N2 amplitude, GBOs, PE, and PCMI were extracted for these trials (Figures 3B–3E). One-way repeated-measures ANOVA revealed significant differences across the four trials for GBOs (F_(3, 66)_ = 3.773, *p* = 0.017, Table S6), PE (F_(3, 66)_ = 5.975, *p* = 0.0011, Table S7), and PCMI (F_(3, 66)_ = 5.386, *p* = 0.0022, Table S8) but not for LEP-N2 amplitude (F_(3, 66)_ = 0.3848, *p* = 0.764, Table S5, Figure 3F). Post-hoc comparisons showed that GBOs could distinguish between NFA2 and FA2 trials (Figure 3G), suggesting a moderate sensitivity to the recovery of anesthesia and nociceptive processing. PE exhibited a slightly higher sensitivity, differentiating NFA2 trials from both FA1 and FA2 trials (Figure 3H). Notably, PCMI demonstrated significant differences between NFA2 and FA1, NFA2 and FA2, as well as NFA1 and FA2 (Figure 3I). Together, these results suggested that GBOs, PE, and PCMI reflect, at least partly, the transition from unresponsive to wakefulness; however, each feature alone provides only limited predictive power for accurately determining anesthesia state shifting.

### Integrated nociceptive-related neural responses discriminate four anesthesia states via unsupervised machine learning

**Figure 4:**
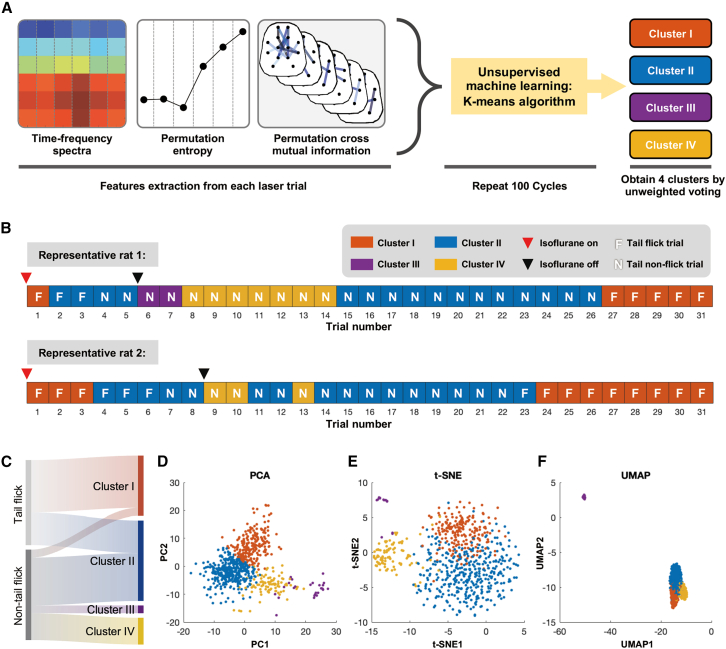
Classification of anesthesia states using unsupervised machine learning based on single-trial nociceptive-evoked neural features (A) Schematic of the analytical pipeline, showing neural feature extraction and subsequent unsupervised machine learning for clustering anesthesia states. (B) Representative clustering results for two rats. (C) Sankey diagram illustrating the distribution of flick and non-flick trials across the four identified clusters. (D–F) Two-dimensional visualizations of the four identified clusters using (D) principal-component analysis (PCA), (E) t-distributed stochastic neighbor embedding (t-SNE), and (F) uniform manifold approximation and projection (UMAP); *n* = 713 trials.

Given that individual nociceptive-related neural features, such as GBOs, PE, and PCMI, showed promising yet imperfect sensitivity in tracking anesthesia states during both induction and emergence phases, we sought to improve state identification by integrating these features into a multivariate representation. Specifically, spectral power, PE, and PCMI were extracted from 14 electrodes within the time window spanning −500 to 1,000 ms relative to stimulus onset for each trial, yielding a high-dimensional feature space of nociceptive-related neural activity. Using this integrated feature set from all 713 trials, we applied an unsupervised *k*-means clustering approach to identify latent structure in the data (Figure 4A). The algorithm partitioned 713 trials into four distinct clusters (Figures 4B–4F), as indicated by the agglomerative hierarchical cluster tree (Figures S1A and S1B). This clustering solution demonstrated high stability across 100 repetitions of independent training (Figures S1C and S1D show all rats together; Figure S2 shows each rat’s clustering results), and leave-one-out stability analysis further confirmed the robustness of the clustering results (Figure S3).

**Figure 5:**
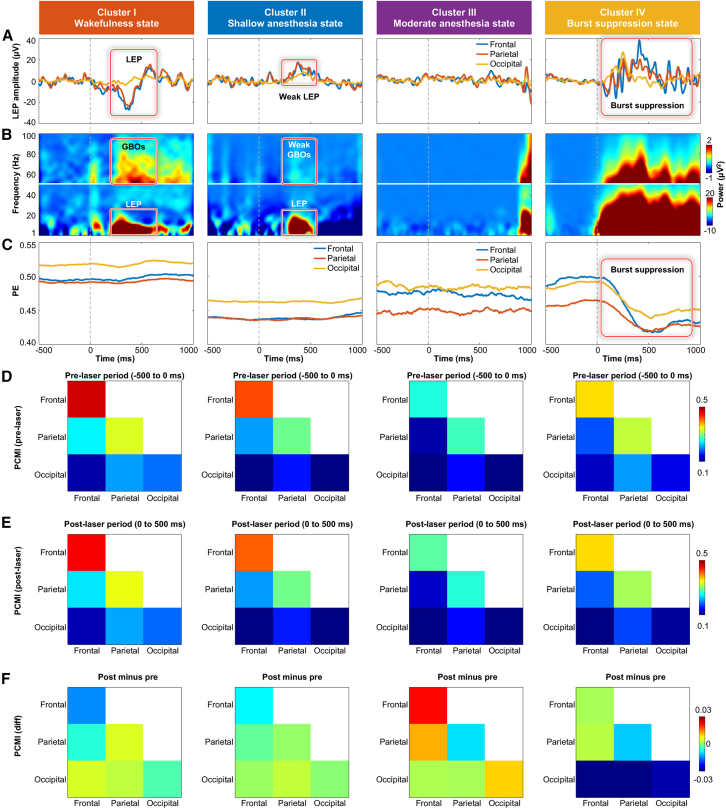
Four anesthesia states identified by unsupervised machine learning (A–C) Group-level (A) LEPs, (B) TFDs, and (C) PE of nociceptive-evoked neural responses for each cluster. (D–F) Group-level of PCMI within (D) pre-stimulus (−500 to 0 ms) and (E) post-stimulus (0–500 ms) intervals, as well as their (F) difference (post-pre), for each cluster. The apparent high-power event around 1,000 ms in cluster 3’s TFD (B) reflects a small number of retained, subthreshold edge artifacts, rather than a reproducible physiological feature. Frontal: FL1, FR1, FL2, and FR2 electrodes; parietal: PL1, PR1, PL2, and PR2 electrodes; occipital: OR and OL electrodes.

To characterize the functional properties of the identified clusters, we computed the laser-evoked potentials (LEPs), time-frequency distributions (TFDs), PE, and PCMI across all trials for each cluster (Figure 5). Cluster 1 exhibited pronounced LEPs and GBOs (Figures 5A and 5B, left column), and consisted predominantly of tail-flick trials (Figure 4C), suggesting an awake state characterized by high sensitivity to external nociceptive stimuli. Cluster 2 showed weak yet discernible LEPs and GBOs (Figures 5A and 5B, second column) following nociceptive laser stimuli. These responses were diminished and delayed compared with the fully awake state,^14^ suggesting some residual nociceptive processing.^24^ In addition, cluster 2 predominantly occurred at the beginning and the end of non-flick trials (Figure 4B) and included both tail-flick and non-flick trials (Figure 4C), indicative of a shallow anesthesia state. Cluster 3 displayed minimal changes in LEPs, TFDs, and PE (Figures 5A–5C, third column), while PCMI patterns resembled those associated with moderate anesthesia state^25^ (Figures 5D–5F, third column), suggesting that this cluster corresponds to a moderate level of anesthesia.^26^ Cluster 4 was distinguished by bursts of high-amplitude fluctuations following nociceptive laser stimuli (Figures 5A and 5B, right column), accompanied by a remarkable decrease in PE (Figure 5C, right column), a hallmark of burst suppression.^27^ Notably, this cluster appeared exclusively after isoflurane cessation (Figures 4B and S2), a time period when burst suppression commonly occurs,^25^ suggesting that laser stimulation can trigger a burst. We also quantified the post-stimulus low-frequency spectral power (1–30 Hz, i.e., ERP), high-frequency spectral power (55–90 Hz, i.e., GBOs), PE, and PCMI (Figure S4; Tables S9–S13) and found significant differences across these four states. Collectively, these results demonstrated that integrating nociceptive-related spectral power, PE, and PCMI features enables the successful identification of four distinct anesthesia states (i.e., awake, shallow anesthesia, moderate anesthesia, and burst suppression) via unsupervised machine learning.

### Complementary information across Spectra, PE, and PCMI supports a more stable integrated clustering solution

**Figure 6:**
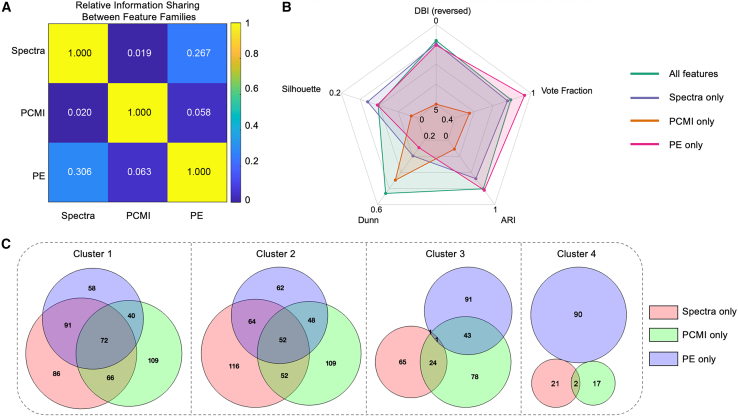
Independent evaluation of feature families and cross-feature structural correspondence (A) Relative information sharing between feature families (spectra, PCMI, and PE), quantified using normalized mutual information. Diagonal elements represent within-feature self-consistency, and off-diagonal elements indicate cross-feature shared information. Low off-diagonal values suggest limited redundancy between feature families. (B) Clustering quality and stability metrics obtained from independent *k*-means clustering using each feature family alone (Spectra only, PCMI only, and PE only) and from the integrated feature set (all features). The metrics include Davies-Bouldin index (DBI, reversed plotted), Silhouette coefficient, Dunn index, adjusted Rand index (ARI), and consensus vote fraction across repeated run. The integrated feature model demonstrates overall improved compactness, separation, and stability compared with individual feature families. (C) Cluster-level overlap among single-feature. For each cluster, Venn diagrams illustrate overlap among clusters obtained using spectral-only, PCMI-only, and PE-only features. Partial overlaps indicate that different feature families capture distinct aspects of the underlying state structure.

To assess potential redundancy among feature families, we quantified cross-family dependence using a mutual information (MI)-based relative information sharing analysis (Figure 6A). This row-normalized matrix revealed limited overlap between feature families: Spectra and PE exhibited moderate coupling (26.7% of Spectra’s internal information and 30.6% of PE’s internal information), whereas PCMI showed minimal overlap with either Spectra (∼2%) or PE (∼6%), and Spectra-PCMI coupling was near-minimal (∼2%). Collectively, these results argue against substantial redundancy and indicate that connectivity-based PCMI contributes largely independent dimensions relative to spectral- and complexity-based features.

We next asked whether these partially independent information sources translate into complementary clustering structure. Using identical preprocessing and *k*-means settings (*k* = 4) as the integrated model, we performed independent clustering using Spectra-only, PE-only, and PCMI-only feature sets and compared them to the all-feature solution by using multiple quality and run-to-run/consensus metrics (Figure 6B; Table S13). Across single-feature solutions, each feature family exhibited measurable discriminative structure; however, performance profiles differed across metrics. Notably, PCMI-only clustering showed weak separability and low agreement across runs, consistent with its role as an independent—but alone insufficient—descriptor of state structure. In contrast, PE-only clustering produced high within-run cohesion (e.g., higher adjusted Rand index [ARI] and vote fraction) but comparatively weaker inter-cluster separation (lower Dunn index), suggesting that PE alone captures a strong global axis of anesthetic modulation yet does not fully resolve the multidimensional subdivision across all four latent states. By integrating Spectra, PE, and PCMI, the all-feature solution provided a more balanced and robust partition.

Finally, to evaluate structural correspondence rather than only scalar clustering scores, we compared cluster memberships within each single-feature solution at the cluster level (Figure 6C). For each cluster, overlap with Spectra-only, PE-only, and PCMI-only clusters was only partial: clusters 1 and 2 showed substantial correspondence across features, whereas clusters 3 and 4 displayed limited overlap and divergent assignments across feature spaces. This pattern indicates that different feature families encode partially distinct aspects of the underlying anesthetic-state manifold and that no single feature family fully reproduces the multidimensional configuration recovered by the integrated representation.

## Discussion

In the present study, we developed an electrophysiological protocol by integrating nociceptive-evoked cortical responses to classify brain states during isoflurane-induced general anesthesia. By extracting laser-evoked cortical features, i.e., GBOs, PE, and PCMI, we evaluated their effectiveness in tracking transitions of nociceptive processing. GBOs and PE more reliably reflected the loss of nociception during induction, whereas PCMI served as a more sensitive indicator of the recovery of nociception during emergence. Integrating these cortical features, we developed an unsupervised machine learning pipeline based on *k*-means clustering to determine anesthesia states in rats. This approach successfully identified four latent states corresponding to distinct phases of anesthesia: awake, shallow anesthesia, moderate anesthesia, and burst suppression. Importantly, these states were identified without behavioral markers, underscoring the potential of nociceptive-evoked cortical responses as objective, label-free biomarkers for real-time brain state monitoring. Beyond methodological innovation, this study extends the utility of pain-related neural indicators, demonstrating that nociceptive signals can be used to effectively capture the analgesic component of anesthesia. The proposed integrated framework provides a theoretical foundation for feature-level stratification in closed-loop systems, paving the way for more precise and autonomous readouts of brain states during general anesthesia.

### Integrating nociceptive features enables reliable anesthesia state identification

Our findings demonstrate that nociceptive-evoked cortical responses can be effectively utilized to detect distinct anesthesia states. Notably, individual features captured certain aspects of anesthesia states, and their performance in detecting anesthesia states was phase dependent and limited when considered in isolation. During the induction phase, GBOs and PE effectively tracked the loss of behavioral responsiveness, whereas LEP-N2 amplitude and PCMI were relatively insensitive. In contrast, during the emergence phase, PCMI emerged as the most discriminative feature, reflecting the progressive re-establishment of large-scale functional connectivity across brain regions, while GBOs and PE exhibited only modest sensitivity in tracking the recovery of behavioral responsiveness. These results likely stem from the intrinsic constraints of each feature: spectral power tends to saturate under deep anesthesia, masking subtle recovery-related signals^28^; complexity measures like PE lose their dynamic range when global neural activity is strongly suppressed^29^; and PCMI, as a network-level metric, requires the reactivation of cortical communication pathways to regain informativeness.^30^

Viewed from a physiological perspective, these phase-dependent effects are expected because each feature family emphasizes a distinct dimension of the anesthesia trajectory. GBO primarily indexes nociceptive processing, closely reflecting the intensity and cortical encoding of stimulus-evoked responses, whereas PE captures global dynamical state changes (i.e., shifts in cortical complexity) that accompany transitions into and out of suppressed brain states. PCMI, in contrast, quantifies inter-regional coupling and, thus, becomes most informative when long-range communication pathways re-emerge and re-stabilize. Because analgesic processing, global cortical dynamics, and network-level coupling do not evolve synchronously across induction and emergence phases, no single feature can fully characterize state transitions across all phases.

To overcome these limitations, we integrated spectral, complexity, and connectivity features into a unified multivariate representation, combining complementary dimensions of neural information—signal energy,^31^ algorithmic complexity,^32^ and network topology.^33^ This composite feature space enabled our clustering model to identify separable brain states with high reliability. Specifically, the integrated features yielded the lowest Davies-Bouldin index (DBI), the highest Dunn index, and the most consistent trial-level cluster assignments, confirming that GBOs, PE, and PCMI provided non-redundant and synergistic contributions to anesthesia state identification. Therefore, by leveraging the multidimensional nature of pain-related neural responses, our approach establishes a scalable framework for stratifying brain states, without relying on behavioral labels.

The key advance of this study is the stratification of anesthesia states using stimulus-evoked, nociception-related electrophysiology, an approach that, to our knowledge, has not been established in prior rodent depth-monitoring work. Most previous studies have focused primarily on spontaneous EEG/ECoG features^1^^,^^2^^,^^3^^,^^25^ or have relied on a single behavioral readout^34^; therefore, they could mainly capture the hypnotic/sedation-related aspect of anesthesia, rather than directly probing nociceptive processing. Importantly, by benchmarking against our previous non-task anesthesia study,^25^ we found that the PCMI signature of the moderate anesthesia state was highly similar between the task and non-task paradigms. This convergence suggests that, under a stable moderate anesthesia state, cortical information integration reaches a suppressed and stable regime that remains largely unchanged, even in the presence of external nociceptive stimulation. In other words, once anesthesia enters this stable state, the brain appears unable to effectively integrate external nociceptive inputs at the network level. Beyond this convergence, the task-based paradigm provides information that cannot be obtained from spontaneous recordings. By incorporating nociceptive-evoked cortical responses, the present framework enables a direct examination of how external noxious inputs interact with different anesthesia states, thereby extending beyond sedation-related state characterization alone. Notably, although burst suppression is a well-established phenomenon during deep anesthesia,^27^ our results further reveal that external stimulation can induce time-locked burst-like activity. Together, these results indicate that our framework both remains consistent with the established anesthesia electrophysiology and extends it by incorporating nociception-linked readouts, offering new insight into the complex neural dynamics underlying anesthetic depth.

### Bridging neural mechanisms and engineering for nociception-based brain state evaluation

In clinics, general anesthesia has three principal components, i.e., analgesia, sedation, and muscle paralysis, each of which is mediated by distinct neural substrates and modulated by different pharmacological agents.^35^ However, current clinical monitoring predominantly targets sedation or loss of consciousness, often overlooking the direct assessment of nociceptive processing.^36^ This oversight is clinically consequential, as analgesia is mechanistically and temporally dissociable from sedation, and incomplete analgesia may result in intraoperative awareness or cortical nociceptive processing despite the absence of overt behavioral responses.^37^ Indeed, our results demonstrated that even in non-flick trials, when nocifensive behaviors were completely abolished, laser-evoked GBOs remained detectable in the cortex. Notably, these GBOs are known to correlate selectively and robustly with the subjective perception of pain^11^ and are considered direct and obligatory neural markers of perceived pain intensity.^13^^,^^17^ Therefore, this persistence indicates that nociceptive information continues to reach cortical circuits independent of motor output, challenging the long-standing reliance on reflexive behaviors, such as the tail flick,^34^ as proxies for pain perception.

From a neuroengineering perspective, the observed dissociation between behaviors (tail flick) and cortical nociceptive responses highlights the need for brain-based biomarkers that enable real-time assessment of nociceptive processing. GBOs, a well-validated biomarker of cortical nociceptive processing,^10^^,^^16^^,^^17^^,^^23^ exhibit a high signal-to-noise ratio with intracranial recordings^38^ and are spatially and spectrally well characterized. By integrating GBOs, which reflect nociceptive processing, with PE, which characterizes neural signal complexity, and PCMI, which captures large-scale functional connectivity, we constructed a multivariate representation spanning complementary dimensions of neural dynamics. Integrating these dimensions with an unsupervised machine learning framework allows for comprehensive and precise stratification of anesthesia states, advancing from behavior-dependent assessments toward label-free, physiologically grounded brain state evaluation.

It should be noted that the choice of *k* = 4 as the optimal number of clusters was based on an empirical decision derived from the current dataset and not an intrinsic theoretical justification. Although *k* = 4 was selected through an initial decision-tree analysis, we further validated this choice by performing a sensitivity analysis, comparing *k* = 3, 4, and 5 using multiple clustering quality and reproducibility metrics, including Silhouette coefficient, DBI, Dunn index, and run-to-run stability (Figure S5). The results from this additional analysis consistently support *k* = 4 as the most balanced solution. While higher values of *k* can always be requested in unsupervised clustering, our data suggest that *k* = 4 represents the best compromise between separation and stability given the current sample size and signal-to-noise characteristics. We acknowledge that with larger and more diverse datasets, a finer partition (e.g., *k* = 5) may become justifiable, but based on the present analysis, *k* = 4 provides the most robust and reproducible clustering solution.

### A flexible framework for scalable EEG-based brain state monitoring

A key advantage of our proposed approach lies in its feasibility for real-time implementation. All nociceptive-related cortical features were extracted from a short peri-stimulus window (−500 to 1000 ms relative to the stimulus onset). The short temporal window enables efficient computation, making real-time implementation on portable or embedded devices feasible.^39^^,^^40^ Such efficiency makes the proposed pipeline well suited for integration into systems or portable monitoring devices with stringent latency and memory constraints.

Although the current feature combination (Spectra + PE + PCMI) yielded robust and stable classification performance, it may not represent the optimal feature set. Future translational applications may benefit from systematic optimization of the feature space. For example, instead of full-spectrum power, focusing on specific frequency bands may provide a more targeted representation of nociceptive dynamics.^17^ Similarly, network-level indicators like PCMI could be replaced or supplemented with more interpretable and computationally efficient alternatives, such as the weighted-phase lag index or graph-theoretical metrics.^31^^,^^41^ Importantly, the goal of the present work was not to replace spontaneous indices but to complement them—leveraging spontaneous activity as a marker of sedation/hypnosis while adding stimulus-locked nociceptive readouts—to better capture the analgesia-related aspect of anesthetic state. This approach enabled a more complete and stratified characterization of anesthetic states, offering an efficient, real-time solution for monitoring multiple facets of anesthesia. Altogether, these considerations suggest the flexibility and scalability of our framework, which not only performs well under experimental conditions but also offers a practical foundation for further engineering optimization in clinical applications.

### Limitations of the study

Several limitations should be acknowledged. First, although the proposed framework holds promise for extending to other anesthetic agents and nociceptive modalities, the current study remains primarily laboratory based, rather than being generalizable for immediate clinical application. While rat ECoG closely parallels human EEG,^10^ further validation is needed to confirm its clinical relevance. Second, the laser-evoked pain model, while valuable for mechanistic insights, may have limited clinical relevance compared to surgery-related nociceptive inputs. Third, although the current model identified four distinct anesthesia states based on 713 trials obtained from 23 male and female rats, the study was not specifically designed or statistically powered to evaluate sex-dependent effects. In addition, the present framework was developed under a relatively restricted set of anesthetic conditions and nociceptive stimuli. Future studies using larger and more balanced cohorts, together with a broader range of anesthetic regimens and nociceptive modalities, will be important for assessing potential sex-related differences, improving model generalizability, and determining whether additional latent brain states can be resolved under more diverse experimental conditions.

Furthermore, while the model offers a framework for stratifying anesthesia states based on nociceptive electrophysiological features, there is no universally accepted gold-standard label for anesthesia depth, particularly in nociception-focused task paradigms. The absence of such a validation metric limits our ability to benchmark directly against traditional supervised models. Additionally, although our clustering approach has demonstrated reproducibility and biological coherence, future studies incorporating pharmacological or behavioral ground-truth proxies will be crucial to strengthen the model’s predictive power and clinical applicability. Lastly, our model focuses on the neural and EEG aspects of anesthesia monitoring, without addressing pharmacological (e.g., target-controlled infusion) or physiological (e.g., minimum alveolar concentration) methods commonly used in clinical practice. As such, a direct comparison with these established methods is not feasible at this stage. While the potential of closed-loop anesthetic delivery is promising, further research is needed to establish its clinical superiority, including the ability to reliably suppress nociceptive responses at lower anesthesia doses.

## Resource availability

### Lead contact

Requests for further information and resources should be directed to and will be fulfilled by the lead contact, Prof. Li Hu (huli@psych.ac.cn).

### Materials availability

This study did not generate new unique reagents.

### Data and code availability

• Data reported in this paper will be shared by the lead contact upon reasonable request.
• Custom MATLAB code used for PCMI analysis, feature extraction, clustering, and auxiliary analyses has been deposited in Zenodo and is publicly available at https://doi.org/10.5281/zenodo.20069771.
• Any additional information required to reanalyze the data reported in this paper is available from the lead contact upon request.

## Acknowledgments

We thank Xiang Li, Zhijie Wang, Xinru Yao, Zhengyu Tang, Mengwen Zhu, and Archer Li for technical support. The project was supported by 10.13039/100007219Beijing Natural Science Foundation (L246074 and JQ22018).

## Author contributions

Conceptualization, F.Z. and L.H.; methodology, F.Z., W.L., R.W., and T.L.; investigation, F.Z. and W.Z.; writing – original draft, F.Z; writing – review & editing, F.Z., X.Z., M.L., L.Y., and L.H.; funding acquisition, L.H.; resources, L.H.; supervision, F.Z. and L.H.

## Declaration of interests

The authors declare no competing interests.

## Declaration of generative AI and AI-assisted technologies in the writing process

During the preparation of this work, the authors used ChatGPT v.5 solely for language editing and improving readability of the manuscript, i.e., for grammar checking and sentence refinement. The authors reviewed and edited the content as needed and take full responsibility for the content of the publication.

## STAR★Methods

### Key resources table

**Table:** 

REAGENT or RESOURCE	SOURCE	IDENTIFIER
**Animal**		

Sprague-Dawley rats	Charles River	RRID:101

**Software**		

MATLAB	MathWorks	RRID: SCR_001622
EEGLAB 2021a	www.sccn.ucsd.edu/eeglab	RRID: SCR_007292
GraphPad Prism	GraphPad Software	V10.5.0 (673)
RStudio	Posit Software	V2024.04.1 + 748
Custom analysis code	https://zenodo.org/records/20069771	https://doi.org/10.5281/zenodo.20069771

### Experimental model and study participant details

#### Animal

The experiment involved 23 adult Sprague-Dawley rats (12 males and 11 females), each weighing between 300 and 400 g.^25^ The animals were housed under a 12-h light-dark cycle with free access to water and food. The experimental procedures were approved by the Ethics Committee at the Institute of Psychology, Chinese Academy of Sciences (#H20050).

### Method details

#### Surgical procedures

The surgical procedures were identical to those described in our previous publications.^25^^,^^31^ Specifically, sixteen holes were drilled into the skull at predetermined stereotaxic coordinates (Figure 1A), without penetrating the dura mater. Sixteen electrodes (stainless steel screws, 1 mm in diameter), including 14 active electrodes, one ground electrode, and one reference electrode, were then inserted and positioned according to established methods.^31^ The electrode leads were connected to a connector module and secured in place with dental cement. After surgery, each rat was housed individually for at least 7 days to recover before electrocorticography (ECoG) recording.

#### Experimental design and ECoG recording

Rats were allowed to acclimate for 10 min before ECoG recording. ECoG data were recorded while the rats were connected to the recording system (Multi Channel Systems, Germany) at a sampling rate of 1000 Hz and allowed to move freely (Figures 1B and 1C). Initially, an infrared Nd:YAP laser (1340 nm wavelength, 4 ms pulse duration, 2 mm beam diameter, 4 J stimulus energy) was applied to the base of the rat’s tail^42^ at 0% isoflurane. The isoflurane concentration was then increased to 5%, and laser stimuli were delivered at a rate of once per minute. After each laser stimulus, the presence or absence of a tail-flick response was assessed according to established criteria.^14^ When the tail-flick response was absent for two consecutive stimuli, laser delivery was halted, and the isoflurane concentration was reduced to 2.5% for 50 to 60 min. Subsequently, isoflurane administration was discontinued, and laser stimuli were again delivered once per minute. Trials in which the laser stimuli did not properly deliver on the tail base were excluded from further analysis. To prevent nociceptor fatigue or potential skin injury, the laser beam was shifted by approximately 5 mm in a random direction after each trial.^10^

#### Animal ECoG data preprocessing

ECoG data were preprocessed using the EEGLAB toolbox (v2025.0.0)^43^ on MATLAB (v2021a), and the original referenced data were used without any re-referencing. The signals were bandpass-filtered between 1 and 100 Hz and notch-filtered at 50 Hz (from 49 to 51 Hz) to remove powerline noise^44^ via the Butterworth filter (*pop_eegfilt* in EEGLAB). The filtered data were then segmented into 3000-ms epochs (1000-ms before and 2000-ms after the stimulus) and baseline-corrected using the pre-stimulus interval. Trials with amplitudes exceeding 500 μV were rejected,^14^ resulting in the removal of a total of nine trials across all animals.

#### Laser-evoked potentials (LEPs)

To extract low-frequency LEP responses, the preprocessed signals were further low-pass filtered at 30 Hz,^45^ and segmented into epochs spanning −500 to 1000 ms relative to stimulus onset. The amplitude of the dominant negative peak (i.e., the LEP-N2 wave) was measured from four central electrodes (i.e., FL2, FR2, PL1, and PR1) for each single trial by calculating the mean value within the region of interest (ROI) corresponding to the LEP-N2 wave (250–400 ms post-stimulus). To illustrate representative LEP waveforms, signals from the four central electrodes of all rats were averaged to obtain group-level LEP waveforms.

#### Time-frequency analysis

To obtain time-frequency distributions (TFDs), we applied a short-time Fourier transform (STFT) to the preprocessed signals (band-pass filtered at 1 to 100 Hz). We computed the TFDs over the −1000 to 2000 ms interval using a 200-ms Hanning window (1–100 Hz), with 1 ms time steps and 1 Hz frequency resolution via *sub_stft* (Hu et al. 2018).^14^ To minimize edge artifacts, we then retained only the central −500 to 1000 ms segment for subsequent analyses. Baseline correction (−400 to −100 ms) was performed on single-trial TFDs using the subtraction procedure.^46^ Nociception-evoked GBOs were quantified by averaging TFD values within the ROI of 55–90 Hz in frequency and 200 to 500 ms in latency.

#### Permutation entropy (PE) and permutation cross mutual information (PCMI) estimation

PE and PCMI were computed for each electrode and trial using the −1000 to 2000 ms peri-stimulus segment; after estimation, we retained only the central −500 to 1000 ms interval for subsequent analyses. PE estimation used the MATLAB *Permutation entropy (fast algorithm)* toolbox (2018, Valentina Unakafova).^47^ The analysis was performed with the following parameters: *m* = 3, *τ* = 1, and *window* = 256. For comparisons in the induction and emergence phases, laser-evoked PE was quantified by averaging PE values within 0–1000 ms post-stimulus across four central electrodes (i.e., PL1, PR1, PL2, and PR2).^25^

PCMI is a measure used to estimate the coupling between two time series. The PCMI between the two vectors X and Y is defined as: *PCMI* = *PE*_*X*_+*PE*_*Y*_-*PE*_*XY*_. PCMI was computed with the following parameters: *m* = 3 and *τ* = 1. For comparisons in the induction and emergence phases, laser-evoked PCMI was quantified as the mean value across four frontal electrodes (i.e., FL1, FR1, FL2, and FR2) within the 0–500 ms post-stimulus window.^25^

#### Unsupervised machine learning

All laser trials were employed for machine learning analyses. The datasets consisted of 416 non-tail-flick trials and 297 tail-flick trials (a total of 713 trials) collected from 23 rats. Each trial included 1134 features extracted from the epoched time window of −500 to 1000 ms. Specifically, TFDs from each electrode were divided into six time bins (250 ms each) and six frequency, i.e., delta (1–4 Hz), theta (4–7 Hz), alpha (7–13 Hz), beta (13–30 Hz), low gamma (30–45 Hz), and high gamma (55–90 Hz),^25^^,^^31^ resulting in 504 features for 14 electrodes, regarding as the spectral features. Additionally, PE features were segmented into the same six time bins, yielding 84 features for 14 electrodes. PCMI features were computed for pairs of electrodes within each of the six time bins, yielding 546 features for 91 electrode pairs. All featues were z-scored before k-means clustering.

To determine the optimal number of clusters, an agglomerative hierarchical cluster tree was constructed, and an unsupervised k-means clustering algorithm (*kmeans* function in MATLAB) was executed for 100 times to stabilize cluster assignments. The final clustering results were obtained through an unweighted voting process across all 100 times.^48^ Specifically, for each laser trial, a voting-based consensus label was determined using unweighted majority voting:

$${\hat{a}}_{i}=mode\left\{a_{i}^{\left(1\right)},a_{i}^{\left(2\right)},\ldots ,a_{i}^{\left(100\right)}\right\}$$

Where ${\hat{a}}_{i}$ is the final consensus cluster label assigned to the *i*-th trial. Clustering stability was evaluated by analyzing the distribution of final cluster counts, which were visualized using histograms.

To address potential label-switching across repeated k-means runs, we aligned the cluster labels to a reference centroid ordering. For each k-means run, we computed the distances between the obtained centroids and a set of predefined reference centroids. The optimal alignment was performed using the Hungarian assignment algorithm, which minimizes the centroid-to-centroid distance, ensuring consistent label mapping across k-means runs. In the absence of the Hungarian algorithm, a greedy approach was employed to match the closest centroids. The final aligned labels were then mapped to the reference label space, and the centroids were reordered to match the reference order. This procedure ensures that cluster labels remain consistent across different runs and that subsequent analyses are not affected by label mismatches.

Subject-level stability of the machine learning model was evaluated via a leave-one-out cross-validation procedure. The dataset was divided into two subsets 23 times, each time using the data from 22 rats for training the model and the data from the omitted rat for testing. For each time, a dendrogram construction and unsupervised clustering procedure were used to determine cluster centroids, which were subsequently used to classify the data of the left-out rat. The clustering results obtained through leave-one-out validation were then compared with those obtained from the full dataset to assess model stability.

To compare how cluster assignments obtained from each single-feature space corresponded to the integrated all-feature solution, we performed a cluster-overlap analysis using Venn diagrams. Specifically, we constructed Venn diagrams based on the final consensus labels from the integrated all-feature solution and from each single-feature solution (Spectra-only, PE-only, and PCMI-only). For each cluster in the all-feature partition (used as the reference), we identified the cluster from each single-feature partition showing the largest membership overlap, and plotted the set intersections to summarize the degree of shared trial/subject membership across solutions.

#### Bootstrap-based validation of cluster number and solution stability

To evaluate the robustness of the selected number of clusters and to avoid over-interpreting an arbitrary choice of k, we performed a subject-level, paired bootstrap validation on the All-features dataset. Specifically, we generated 1000 bootstrap resamples by sampling subjects with replacement from the original cohort. For each bootstrap sample and each candidate number of clusters (k = 3, 4, and 5), we performed k-means clustering 100 times with different random initializations. To address label switching across repeated runs, we aligned cluster identities to a reference labeling, which was determined based on the run with the best within-cluster sum of distances. After alignment, we derived a consensus label for each subject by majority voting across the 100 aligned runs.

For each bootstrap replicate and each k, we quantified clustering quality and reproducibility using complementary metrics computed on the consensus labels: Davies–Bouldin index (DBI; lower is better), mean Silhouette coefficient (higher is better), and Dunn index (higher is better). We additionally quantified solution stability using a subject-level vote fraction, defined as the proportion of the 100 aligned runs that assigned a subject to its consensus cluster; stability was summarized as the median vote fraction across subjects for each bootstrap replicate. Bootstrap distributions (median and 2.5th–97.5th percentiles) were used to summarize metric uncertainty, and paired bootstrap comparisons were used to compare k = 4 versus k = 3 and k = 5.

#### Assessment of clustering quality

To evaluate the effectiveness of feature integration in improving clustering performance, three quantitative indices were computed for each repetition to assess clustering quality, i.e., the DBI, Silhouette coefficient, and Dunn indexes.^49^^,^^50^ In addition, trial-wise clustering stability was quantified at the single-trial level by computing the proportion of repetitions in which its assigned label matched the final consensus label. Run-to-run reproducibility was quantified using the adjusted Rand index (ARI). Specifically, after aligning cluster labels to a reference labeling to remove label switching, we computed the ARI between each run’s partition and the reference partition (or equivalently, between each run and the final consensus partition). ARI ranges from 0 (chance-level agreement) to 1 (identical partitions). For each trial, we calculated the vote fraction as the proportion of the 100 aligned k-means runs that assigned it to its final consensus cluster (i.e., the majority-vote label). The overall vote fraction was then summarized across all trials, and we report both the median vote fraction (median across trials) and the distribution (e.g., interquartile range, IQR) to describe clustering stability.

#### Relative information sharing analysis

To quantify the degree of redundancy between feature families (Spectra, PCMI, and PE), we estimated relative information sharing based on mutual information (MI). For each feature family, the trial-by-feature matrix was first standardized (z-scored across trials) and subjected to principal component analysis (PCA). The first three principal components (PC1 to PC3), representing the dominant variance structure within each feature family, were retained to construct a three-dimensional embedding for each trial. This dimensionality reduction step mitigates bias associated with high-dimensional density estimation. Mutual information between multivariate continuous variables was estimated using the Kraskov k-nearest neighbor estimator (k = 5). For each pair of feature families *i* and *j*, cross-family mutual information *I*(*i*,*j*) was computed between their respective three-dimensional embeddings. To provide an internal reference scale for each feature family, within-family information coupling *I*(*i*,*i*) was defined as the mutual information between two overlapping subspaces of its three-dimensional embedding ([PC1, PC2] versus [PC2, PC3]). This quantity reflects internal information coupling rather than self-information. Relative information sharing was then defined as:

$$R_{i\to j}=\frac{I\left(i,j\right)}{I\left(i,i\right)}$$

where $R_{i\to j}$ quantifies the proportion of feature family *i*’s internal information that is shared with feature family *j*. By construction, $R_{i\to i}$ = 1, and the resulting matrix is asymmetric. This normalization yields a scale-invariant index of cross-family information overlap relative to internal coupling strength, enabling direct assessment of feature redundancy.

### Quantification and statistical analysis

Statistical analyses were conducted on single-trial data using Prism (v8.3.1, GraphPad) and MATLAB. Prior to statistical analysis, data normality was assessed for all variables using the Shapiro-Wilk test. For within-subject comparisons across experimental conditions, N2 amplitude, GBO magnitude, PE, and PCMI were analyzed using one-way repeated-measures analyses of variance (RM ANOVA). When significant main effects were detected, post hoc pairwise comparisons were performed using paired-sample t tests with Bonferroni correction to control for multiple comparisons. For comparisons of post-stimulus ECoG measures across the four derived clusters (state-dependent differences), group differences were evaluated using one-way ANOVA (non–repeated-measures). When significant main effects were observed, post hoc pairwise comparisons were conducted using independent-samples t tests with Bonferroni correction.
