# Supplementary material for: Integrating multidimensional nociceptive-related cortical features for unsupervised assessment of anesthesia states in rats
Source: iScience. 2026 May 28;29(6):116152. doi: 10.1016/j.isci.2026.116152 (PMC13233798; doi:10.1016/j.isci.2026.116152)
Supplement: Document S1. Figures S1–S5 and Tables S1–S14 [file mmc1.pdf]

iScience, Volume *29*

Supplemental information

Integrating multidimensional nociceptive-related cortical features for unsupervised assessment of anesthesia states in rats

Fengrui Zhang, Wenqian Zhou, Wen Liang, Ruoyu Wang, Libo Zhang, Xiao Zhang, Meizi Liu, Tong Li, Lupeng Yue, and Li Hu

Figure S1. Clustering of all single trials using unsupervised machine learning.

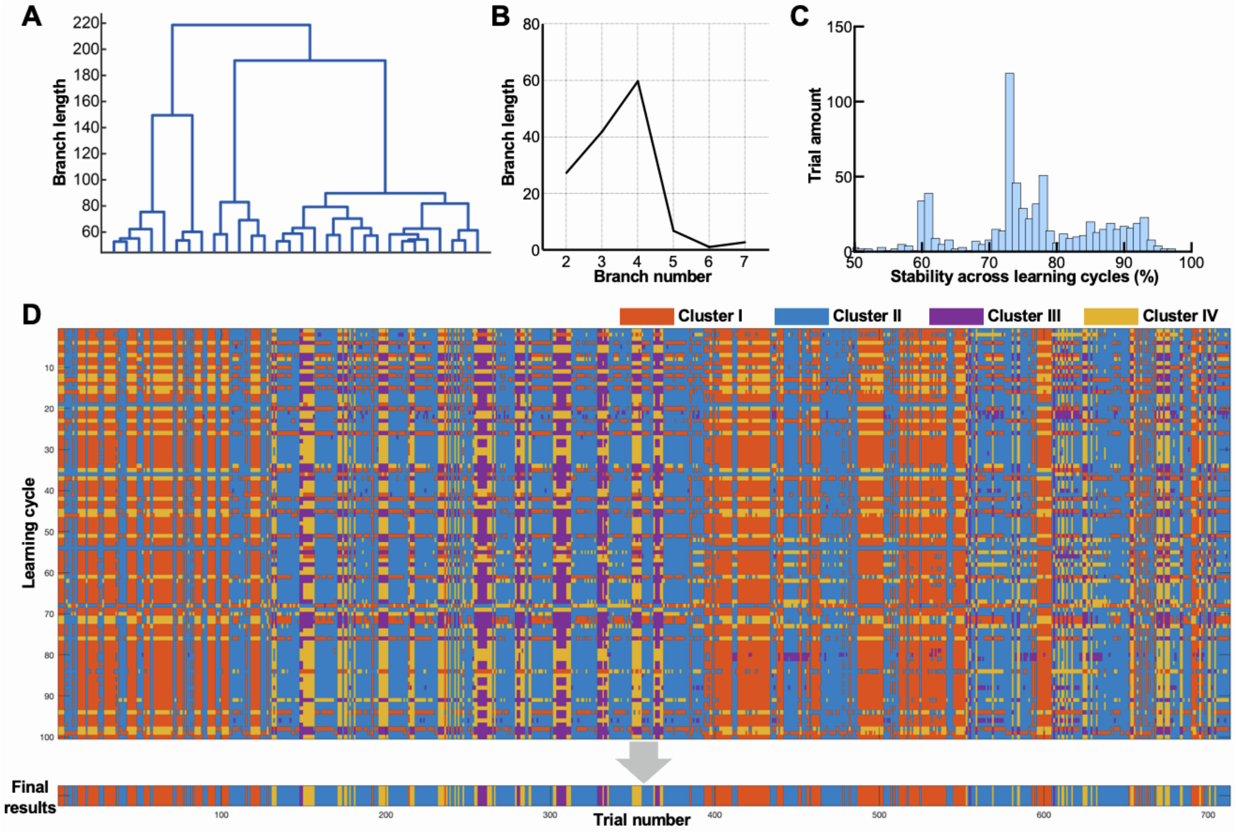

(**A, B**) Cluster dendrogram (A) and length of branches (B) illustrating the hierarchical organization of the data, revealing four distinct clusters. (**C**) Trial-wise clustering stability across 100 learning cycles. (**D**) Clustering results obtained from 100 iterations of k-means clustering (top). Rows indicate individual clustering iterations, and columns indicate trials. The final clustering result was derived by majority voting across the 100 iterations (bottom). Related to Figure 4.

Figure S2. Per-subject chronological summary of cluster assignments across the anesthesia course

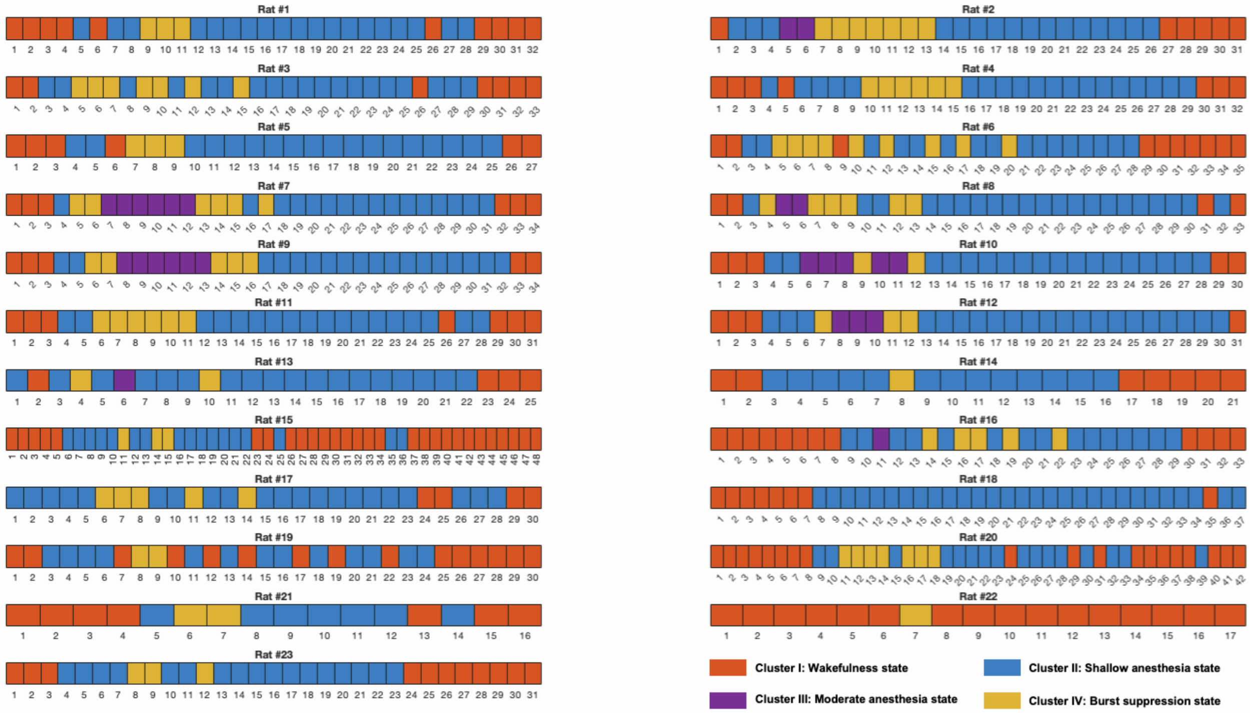

Each row represents one rat, and each colored block denotes the cluster identity assigned to an individual trial (or trial-level summary window). Trials within each rat are displayed in chronological order along the experimental session to preserve the temporal progression of the anesthesia course (from earlier to later time points). Colors indicate the four clusters (Cluster 1 to 4), corresponding to distinct anesthetic-state patterns identified by the unsupervised model. Related to Figure 4.

Figure S3. Leave-one-out cross-validation.

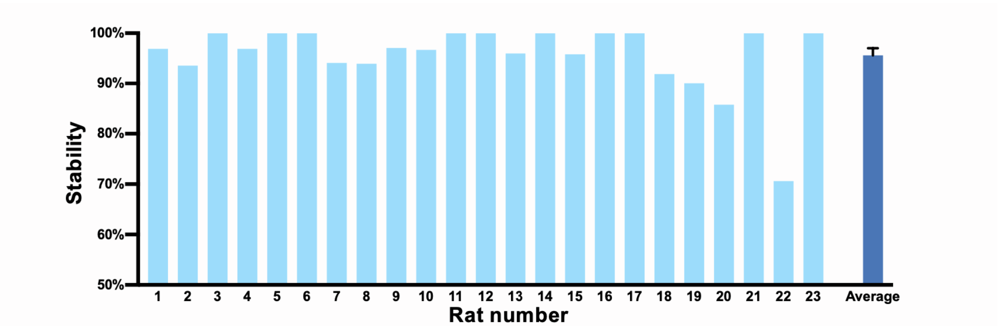

The stability of clustering results was assessed through leave-one-out cross-validation at subject level. Related to Figure 4.

Figure S4. State-dependent differences in post-stimulus ECoG measures across the four derived clusters.

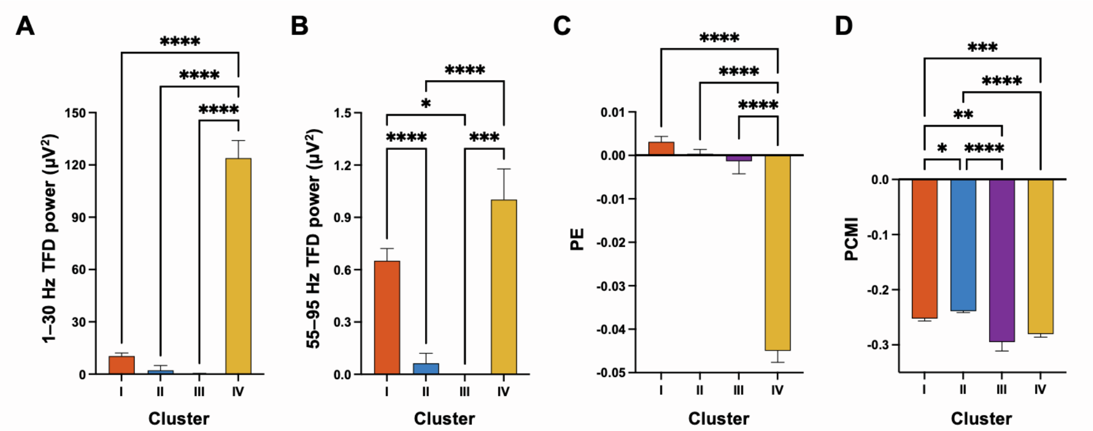

Bar plots show the baseline-corrected change (Δ) for each measure, computed as: mean value within 0–800 ms after laser stimulation minus the mean value in the pre-stimulus baseline window. (**A**) Low-frequency power (1–30 Hz; TFD power, from central electrodes). (**B**) High-frequency power (55–90 Hz; TFD power, from central electrodes). (**C**) Whole-brain PE, averaged across all electrodes. (**D**) Whole-brain PCMI, averaged across all electrode pairs. Sample sizes: Cluster I, n=211; Cluster II, n=382; Cluster III, n=25; Cluster IV, n=94. All values are presented as mean ± SEM. Group differences were assessed using one-way ANOVA followed by Bonferroni's post hoc multiple-comparison tests; horizontal brackets indicate significant pairwise comparisons (*p<0.05, **p<0.01, ***p<0.001, ****p<0.0001). Related to Figure 5.

Figure S5. Comparison of clustering quality and stability across candidate k values

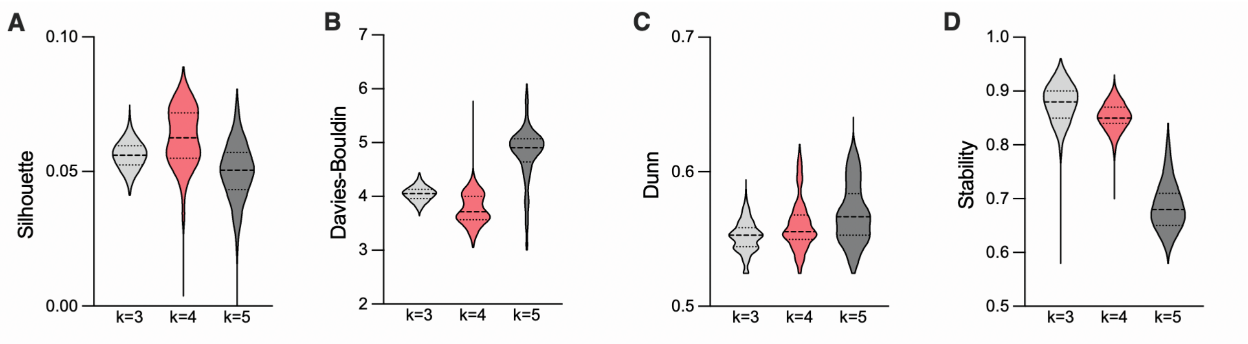

(A) Silhouette coefficient, (B) Davies–Bouldin index (DBI), (C) Dunn index, and (D) clustering stability across repeated runs. Each violin plot represents the distribution of run-level estimates obtained from repeated clustering initializations.

Table S1. Statistical results for Figure 2H

One-way RM ANOVA	SS	DF	MS	F (DFn, DFd)	P Value
Between groups	16404	3	5468	F (3, 66) = 1.422	0.244
Within group	99694	22	4532	F (22, 66) = 1.179	0.297
Residual	253724	66	3844		
Total	369823	91			

Table S2. Statistical results for Figure 2I

One-way RM ANOVA	SS	DF	MS	F (DFn, DFd)	P Value
Between groups	59.64	3	19.88	F (3, 66) = 17.39	<0.001
Within group	45.13	22	2.051	F (22, 66) = 1.794	0.036
Residual	75.46	66	1.143		
Total	180.2	91			

Post Hoc Comparisons	Mean Diff.	95.00% CI of diff.	Adjusted P Value
F2 vs. F1	1.316	0.4584 to 2.174	0.0015
F2 vs. NF1	1.914	1.056 to 2.771	0.0004
F2 vs. NF2	2.026	1.168 to 2.884	0.0001
F1 vs. NF1	0.5977	-0.2600 to 1.455	0.0178
F1 vs. NF2	0.7098	-0.1478 to 1.568	0.0019
NF1 vs. NF2	0.1122	-0.7455 to 0.9699	0.5449

Table S3. Statistical results for Figure 2J

One-way RM ANOVA	SS	DF	MS	F (DFn, DFd)	P Value
Between groups	0.2480	3	0.08268	F (3, 66) = 209.7	<0.0001
Within group	0.05333	22	0.002424	F (22, 66) = 6.147	<0.0001
Residual	0.02603	66	0.0003943		
Total	0.3274	91			

Post Hoc Comparisons	Mean Diff.	95.00% CI of diff.	Adjusted P Value
F2 vs. F1	0.1210	0.1051 to 0.1369	<0.0001
F2 vs. NF1	0.1219	0.1060 to 0.1378	<0.0001
F2 vs. NF2	0.04539	0.02946 to 0.06132	<0.0001
F1 vs. NF1	0.0008912	-0.01504 to 0.01682	>0.9999
F1 vs. NF2	-0.07562	-0.09155 to -0.05969	<0.0001
NF1 vs. NF2	-0.07651	-0.09244 to -0.06058	<0.0001

Table S4. Statistical results for Figure 2K

One-way RM ANOVA	SS	DF	MS	F (DFn, DFd)	P Value
Between groups	0.02429	3	0.008096	F (3, 66) = 3.447	0.0215
Within group	0.4772	22	0.02169	F (22, 66) = 3.502	<0.0001
Residual	0.4088	66	0.006193		
Total	0.9102	91			

Post Hoc Comparisons	Mean Diff.	95.00% CI of diff.	Adjusted P Value
F2 vs. F1	-0.02998	-0.09311 to 0.03314	>0.9999
F2 vs. NF1	-0.02642	-0.08954 to 0.03671	>0.9999
F2 vs. NF2	0.007516	-0.05561 to 0.07064	>0.9999
F1 vs. NF1	0.003566	-0.05956 to 0.06669	>0.9999
F1 vs. NF2	0.03750	-0.02562 to 0.1006	0.6652
NF1 vs. NF2	0.03394	-0.02919 to 0.09706	0.8904

Table S5. Statistical results for Figure 3F

One-way RM ANOVA	SS	DF	MS	F (DFn, DFd)	P Value
Between groups	2537	3	845.7	F (3, 66) = 0.3848	0.764
Within group	82814	22	3764	F (22, 66) = 1.713	0.049
Residual	145032	66	2197		
Total	230383	91			

Table S6. Statistical results for Figure 3G

One-way RM ANOVA	SS	DF	MS	F (DFn, DFd)	P Value
Between groups	2.039	3	0.6796	F (3, 66) = 3.773	0.017
Within group	8.906	22	0.4048	F (22, 66) = 2.248	0.006
Residual	11.89	66	0.1801		
Total	22.83	91			

Post Hoc Comparisons	Mean Diff.	95.00% CI of diff.	Adjusted P Value
NFA2 vs. NFA1	-0.1797	-0.4712 to 0.1118	0.5265
NFA2 vs. FA1	-0.2165	-0.5776 to 0.1445	0.5768
NFA2 vs. FA2	-0.4191	-0.7754 to -0.06281	0.0151
NFA1 vs. FA1	-0.03686	-0.4066 to 0.3328	>0.999
NFA1 vs. FA2	-0.2394	-0.6376 to 0.1587	0.5717
FA1 vs. FA2	-0.2026	-0.5925 to 0.1873	0.8778

Table S7. Statistical results for Figure 3H

One-way RM ANOVA	SS	DF	MS	F (DFn, DFd)	P Value
Between groups	0.006620	3	0.002207	F (3, 66) = 5.975	0.0011
Within group	0.06182	22	0.002810	F (22, 66) = 7.608	<0.0001
Residual	0.02438	66	0.0003694		
Total	0.09282	91			

Post Hoc Comparisons	Mean Diff.	95.00% CI of diff.	Adjusted P Value
NFA2 vs. NFA1	-0.009228	-0.02464 to 0.006188	0.6494
NFA2 vs. FA1	-0.01906	-0.03447 to -0.003640	0.0077
NFA2 vs. FA2	-0.02133	-0.03674 to -0.005913	0.0022
NFA1 vs. FA1	-0.009828	-0.02524 to 0.005588	0.5253
NFA1 vs. FA2	-0.01210	-0.02752 to 0.003315	0.2188
FA1 vs. FA2	-0.002272	-0.01769 to 0.01314	>0.9999

Table S8. Statistical results for Figure 3I

One-way RM ANOVA	SS	DF	MS	F (DFn, DFd)	P Value
Between groups	0.1116	3	0.03718	F (3, 66) = 5.386	0.0022
Within group	1.079	22	0.04904	F (22, 66) = 7.103	<0.0001
Residual	0.4557	66	0.006904		
Total	1.646	91			

Post Hoc Comparisons	Mean Diff.	95.00% CI of diff.	Adjusted P Value
NFA2 vs. NFA1	-0.006149	-0.07280 to 0.06050	>0.9999
NFA2 vs. FA1	-0.06813	-0.1348 to -0.001478	0.0424
NFA2 vs. FA2	-0.07653	-0.1432 to -0.009882	0.0159
NFA1 vs. FA1	-0.06198	-0.1286 to 0.004671	0.0829
NFA1 vs. FA2	-0.07038	-0.1370 to -0.003733	0.0328
FA1 vs. FA2	-0.008404	-0.07505 to 0.05824	>0.9999

Table S9. Statistical results for Figure S4A

One-way ANOVA	SS	DF	MS	F (DFn, DFd)	P Value
Between groups	1165468	3	388489	F (3, 709) = 129.1	<0.0001
Residual	2133956	709	3010		
Total	3299424	712			

Post Hoc Comparisons	Mean Diff.	95.00% CI of diff.	Adjusted P Value
Cluster I vs. II	8.083	-4.035 to 20.20	0.3152
Cluster I vs. III	10.22	-19.14 to 39.58	0.8068
Cluster I vs. IV	-113.6	-131.1 to -96.08	<0.0001
Cluster II vs. III	2.137	-26.50 to 30.77	0.9975
Cluster II vs. IV	-121.7	-137.9 to -105.4	<0.0001
Cluster III vs. IV	-123.8	-155.1 to -92.51	<0.0001

Table S10. Statistical results for Figure S4B

One-way ANOVA	SS	DF	MS	F (DFn, DFd)	P Value
Between groups	93.61	3	31.20	F (3, 709) = 23.05	<0.0001
Residual	960.0	709	1.354		
Total	1054	712			

Post Hoc Comparisons	Mean Diff.	95.00% CI of diff.	Adjusted P Value
Cluster I vs. II	0.5881	0.3311 to 0.8451	<0.0001
Cluster I vs. III	0.6501	0.02729 to 1.273	0.0369
Cluster I vs. IV	-0.3504	-0.7219 to 0.02122	0.0728
Cluster II vs. III	0.06197	-0.5454 to 0.6693	0.9936
Cluster II vs. IV	-0.9385	-1.283 to -0.5935	<0.0001
Cluster III vs. IV	-1.000	-1.664 to -0.3365	0.0007

Table S11. Statistical results for Figure S4C

One-way ANOVA	SS	DF	MS	F (DFn, DFd)	P Value
Between groups	0.1754	3	0.05847	F (3, 709) = 149.1	<0.0001
Residual	0.2780	709	0.0003920		
Total	0.4534	712			

Post Hoc Comparisons	Mean Diff.	95.00% CI of diff.	Adjusted P Value
Cluster I vs. II	0.002719	-0.001655 to 0.007092	0.3788
Cluster I vs. III	0.004447	-0.006151 to 0.01504	0.7016
Cluster I vs. IV	0.04807	0.04175 to 0.05439	<0.0001
Cluster II vs. III	0.001728	-0.008606 to 0.01206	0.9732
Cluster II vs. IV	0.04535	0.03948 to 0.05122	<0.0001
Cluster III vs. IV	0.04362	0.03232 to 0.05492	<0.0001

Table S12. Statistical results for Figure S4D

One-way ANOVA	SS	DF	MS	F (DFn, DFd)	P Value
Between groups	0.1882	3	0.06275	F (3, 709) = 20.85	P<0.0001
Residual	2.134	709	0.003010		
Total	2.322	712			

Post Hoc Comparisons	Mean Diff.	95.00% CI of diff.	Adjusted P Value
Cluster I vs. II	-0.01345	-0.02590 to -0.001001	0.0263
Cluster I vs. III	0.04260	0.01243 to 0.07277	0.0012
Cluster I vs. IV	0.02817	0.01017 to 0.04617	0.0002
Cluster II vs. III	0.05605	0.02663 to 0.08547	<0.0001
Cluster II vs. IV	0.04163	0.02491 to 0.05834	<0.0001
Cluster III vs. IV	-0.01443	-0.04659 to 0.01774	>0.9999

Table S13. Clustering Quality and Run-to-Run Stability Metrics for the Integrated All-Feature Representation

Metric	All Features	Spectra only	PCMI only	PE only
DBI (Mean±SD)	2.6379 ±0.7904	2.8229±0.3502	6.6304±1.5109	2.9367±0.6064
DBI (Median / IQR)	2.4194 / 1.0603	2.7288 / 0.4512	6.9284 / 2.3504	2.1289 / 0.9044
Silhouette (Mean±SD)	0.0929±0.0414	0.1305±0.0746	0.0156±0.0153	0.1748±0.0168
Silhouette (Median / IQR)	0.0992 / 0.0355	0.1297 / 0.0502	0.0143 / 0.0077	0.1793 / 0.0079
Dunn (Mean±SD)	0.5304±0.0101	0.2978±0.0260	0.4481±0.0349	0.2466±0.0028
Dunn (Median / IQR)	0.5348 / 0.0103	0.3050 / 0.0523	0.4399 / 0.0373	0.2473 / 0.0031
ARI (Mean±SD)	0.7547±0.1982	0.5969±0.2253	0.1386±0.1151	0.7764±0.1570
ARI (Median / IQR)	0.5851 / 0.3563	0.5473 / 0.3660	0.1205 / 0.1393	0.7425 / 0.0822
Vote Fraction (Mean±SD)	0.8404±0.1599	0.8147±0.1240	0.5135±0.1041	0.9495±0.0903
Vote Fraction (Median / IQR)	0.9000 / 0.2700	0.8300 / 0.1825	0.5000 / 0.1400	1.0000 / 0.1000

Table S14. Bootstrap-based comparison of clustering indices for k selection

(**A**) k = 4 vs k = 3

Metric	Δ (k4 − k3)	95.00% CI	Interpretation
DBI	−0.3720	[−0.8084, −0.0130]	Favors k=4
Silhouette	0.0034	[−0.0192, 0.0231]	Comparable
Dunn	0.0000	[−0.0139, 0.0578]	Comparable
Stability	−0.0300	[−0.1100, 0.0700]	Comparable

(**B**) k = 4 vs k = 5

Metric	Δ (k4 − k5)	95.00% CI	Interpretation
DBI	−1.1335	[−1.8121, 0.4785]	Favors k=4
Silhouette	0.0136	[−0.0155, 0.0375]	Comparable
Dunn	0.0000	[−0.0569, 0.0206]	Comparable
Stability	0.1700	[0.0500, 0.2500]	Favors k=4
